# Monthly Digest

**Published:** 1889-02

**Authors:** 


### MONTHLY DIGEST.

### The Trend of Dental Thought as Presented by our Contemporaries.

CONTINUED FROM DECEMBER NUMBER, PAGE 41.

### AMALGAMS.

In the discussion of a paper read by Dr. A. H. Fuller, before the Missouri State Dental Association, as reported in the *Archives*, Dr. Patterson said he did not think there can be any injurious effects from amalgam after it has hardened and oxides are formed, though he objects to the color in the mouth and uses it only from the pecuniary aspect.

He thinks there can be no poisonous effect from copper amalgam as the oxide is inert, there not being sufficient acetic acid in the mouth to act upon it.

Dr. Pierce thinks that copper salts will be formed by the acidulous moisture in the mouth, and these salts are poisonous. As to the toxic effects, he advocates the idea that the finer the division the greater the effect.

Dr. Conrad thinks that the only danger in copper amalgam lies in the fact that it makes such a perfect filling that we are in danger of putting in too many.

Dr. Patterson does not think it proved that it has any poisonous effects in the mouth. Nitric acid will destroy teeth out of the mouth, yet, though existing in the mouth it does not destroy teeth there.

Nitro-muriatic acid will dissolve gold, yet, existing in the mouth, it does not destroy gold fillings there.

Dr. Fuller spoke of constitutional idiosyncracies in this connection. Some persons cannot endure mercury.

Dr. Patterson replied that so also some persons cannot bear morphine, others quinine, etc., but these remedial agents are not abandoned for that reason.

DENTAL CARIES.

In the Union Meeting of the 5th, 6th, 7th, and 8th District Dental Societies of New York, Dr. I. C. Curtis read a paper reported in the *Cosmos*, on the knowledge of chemistry necessary to the dentist, especially chemical acid reaction in the disintegration of tooth-substance, and the practical means of combating this. The dentist of the future will rely on self-cleansing spaces, antiseptics and alkaline washes, rather than on manipulative skill.

In the discussion of this paper, Dr. Barrett, while admitting the correctness of the chemical ideas laid down, said that the acids named are so diluted in the mouth that the reactions do not take place as claimed. The solution of the question lies in our more recently acquired knowledge of fermentative organisms. The acids which they produce are the cause of caries, as demonstrated by Dr. Miller. Fermentation lies at the very foundation of dental chemistry.

Dr. Dwinelle considers that Dr. Barrett struck the key-note of the subject. Fermentation will account for much in the phenomena of caries that chemistry fails to reveal.

Dr. J. B. Willmott, Toronto, Canada, agrees with Dr. Barrett in regard to fermentation, and cannot believe that acids which are innocuous to the soft tissues of the mouth can destroy well-developed tooth tissue. He thinks that nascent acids only, formed in actual contact with the teeth, can act upon them.

Dr. Brophy takes issue with the statement of the paper that decay does not occur in alkaline mouths, some of the most extreme cases of decay being found in mouths with the stringy saliva which accompanies the alkaline condition.

Dr. Curtis replied to this that the litmus test will show acid conditions in the location of decay, though the general fluids may be alkaline.

Dr. E. T. Darby reconciles the difference of opinion among the speakers, by the fact that they reason from different standpoints. The various theories advanced all have some foundation in fact, the germ or fermentation theory of Dr. Miller alone, however, accounting for all the features of caries.

### EROSION.

The *Cosmos* reports the discussion of the subject of Erosion, in Pennsylvania Odontological Society by Drs. Faught, Truman, and Kirk.

Dr. Faught has tried trimming away all the softened dentine with burrs and polishing the surface.

Dr. Truman would do no cutting, but polishes the surfaces with a stick and pumace, prescribing lime water as a wash, and a chalk paste laid under the lip at night. He believes that the work of destruction is done at night, during the general subsidence of the salivary secretion which, through the day, dilutes the acid secretion of the mucous membrane lining the lips.

### EOVLUTION OF DENTISTRY.

Dr. C. Cutler Smith, Ilian, N. Y., in a brief article in the *Independent Practitioner* traces the evolution of dentistry thus—the first step was the extraction of teeth; the second, repairing diseased teeth or replacing them by artificial substitutes; the third, crown and bridge work by which the natural roots are utilized; fourth, *implantation* by which a sound tooth in a healthy socket takes its place—not as an adopted member of the family, but as an own child. Even should implanted teeth last but two or three years, he thinks the patient would be well repaid, while, should the operation prove permanent he considers that dental science will have taken one of the mightiest strides in its history.

### BICHLORIDE OF MERCURY.

In the *Cosmos*, page 581, Dr. C. J. Essig points out the fact that while, in implantation and similar operations bichloride of mercury is without an equal as a sterilizer, it will not prove available under all conditions, as it must be kept from contact with metals. For example, in the treatment of a root canal, if carried past gold filling the mercuric bichloride would be instantly decomposed by electrolysis, the free mercury deposited on the filling, only an inert fluid reaching the root canal. The same is true of bichloride as a constituent in a mouthwash; contact with gold or amalgam fillings would decompose the sterilizing agent.

PATHOGENIC BACTERIA.

The *Archives* reproduces an editorial from the *British Journal of Dental Science*, on the importance of a knowledge of this subject. The writer cites from Netter's investigations the fact that even in the saliva of numerous ostensibly healthy persons, a bacillus exists which closely resembles the streptoccus pyogenes, a micro-organism associated with, if not the cause of suppurative inflammation. When the buccal epithelium is intact, this organism is unable to do any damage, but with the least abrasion, suppuration will ensue. In many dental operations the lymph channels of the soft tissues, and even the bone centers are opened up, when the results may be serious. The many reported cases of pyæmia following tooth-extraction are doubtless cases in point, and call for closer study and more efficient prophylactic treatment. Antiseptic mouth washes should be freely used, instruments most thoroughly cleansed to avoid the ever-present danger of transferring pathogenic bacteria from one mouth to another.

COCAINE.

The *Archives* also publishes a translation from the *Revue Scientifique* giving numerous cases of serious results from the use of cocaine.

Also a translation of a lecture delivered at the meeting of Natural Scientists and Physicians, at Wiesbaden, by Dr. Telschow, of Berlin, giving the very satisfactory results of the combination of carbolic acid and cocaine in sub-gingival injections for extraction.

The combination consists of from 4 to 8 centigrams of cocaine dissolved in from 10 to 20 drops of water carbolized in the proportion of $\frac{1}{2}$ to 100. The carbolic acid cauterizes the sub-mucous membrane preventing its absorption, thus rendering its action entirely local. Dr. Telschow lays down the following principles:

1st. Make the injection near the margin of the gum because there are very few nerves and lymphatic vessels there.

2nd. Inject in both the lingual and buccal surfaces.

3rd. Separate the gum from the tooth after the cocaine has acted for two minutes and apply a 20 per cent. solution between the tooth and gum. The operation is painless and gives the patient confidence.

### TOOTHACHE DROPS.

In the same journal Dr. J. B. Newby relates a case of very alarming results from the use of certain "Toothache drops" according to the printed instructions, and calls upon the profession to use its influence in securing the enactment of laws preventing the indiscriminate sale of such deadly drugs, the sample in question having been found to contain morphine and ether sufficient to cause the death of several grown persons.

### PHYSIOLOGICAL ASSIMILATION.

Dr. J. B. Lawrence, in the New York Odontological Society, read a paper on this subject, of which only the discussion is given in the *Cosmos* of December. Dr. Lawrence said that he begun the study of the subject fifteen years ago, resorting to chemical analysis, under the late Prof. Draper. The conclusions then reached were that softened dental and osseous tissues showing deficiency in lime salts, were only one of the symptoms of genuine physiological aberrations, and that the use of so-called "Vitalized phosphates" would often aggravate the symptoms, analysis of the excretions showing them to have passed the economy unassimilated. The practice is also unscientific and empirical. Dr. Lawrence indicates the last edition of Gross as epitomizing the theory, and reflecting the views of standard authors.

Dr. V. H. Jackson and Dr. Dwinelle on the other hand, spoke strongly in favor of the use of whole wheat grain, phosphate-feeding, and the use of lime water, considering the practical results of actual practice of more value than the theories even of standard authors. Dr. Dwinelle believes that the character of young patient's teeth may be perceptably changed in a few months time by this mode of treatment.

### NEURALGIA.

Dr. V. H. Jackson, in the First District Dental Society, New York, gave the treatment also reported in the *Cosmos* of a case of periodic neuralgia, complicated with pyorrhea alveolaris and a dead molar. The extraction of the molar was followed by severe secondary hemorrhage three days later, which was controlled by the use of tannic acid on a moist pledget of cotton wedged in the socket. The surgical treatment for pyorrhea alveolaris was supplemented by constitutional treatment for neuralgia, viz: the exhibition of anti-febrin and Monsette's pills, the latter a Paris anti-neuralgic compound containing 1-5 milligramme aconite and 5 centigrammes quinine.

FOUL BREATH.

Dr. L. Milliron, Kimball, Dak., called attention to this subject in the *Archives*, as one deserving more attention than it has received.

Among the causes mentioned are calcareous deposits, caries, ulcers and abscesses, catarrh, diseases of the lungs or stomach, and torpor of the liver.

According to the cause, the treatment must be either mechanical, operative, or medicinal. After removal of calcareous deposits Dr. Milliron prescribes a mouthwash containing sulphur, prepared chalk, and rectified spirits, flavored with peppermint—used with a toothbrush morning and evening.

Decayed or pulpless teeth are, of course, all to be put in order.

If the patient is not referred to an M.D. for constitutional treatment Dr. Milliron advises:

For catarrh—sanguinarea Canadensis; for the stomach—carbolic acid, 5 drops to the ounce of water, or the white of an egg beaten up in a cup of milk; for the liver—leptandra Virginica in from $\frac{1}{2}$ to 1 grain doses, in capsules, night and morning.

CARE OF CHILDREN'S TEETH.

Dr. E. J. Church, Laporte, in a paper read before the Indiana State Dental Society, and reported in the *Ohio Journal*, dwells upon the importance of impressing upon parents the absolute necessity of maintaining a constant condition of cleanliness of the teeth of children—the average parent knowing little about the teeth and manifesting an indifference that is truly appalling. Practical instructions are given for the judicious home-care of the teeth, beginning with the first two incisors, and also the best methods of dealing with the little ones in the dental office.

Among the miscellaneous topics discussed we find as the leading article in the *Cosmos*, a paper on

DENTAL LEGISLATION

Sympathizing with Dr. Shumway in his solitary stand against dental legislation (see *Cosmos* Jan. '88 p. 51). Dr. G. S. Dean, San Francisco, Cal., comes to his support in an elaborate eighteen-page article, in which he seeks to reach a basis for the rational discussion of the subject. The present system is condemned *in toto* as resting on a flimsy basis of superficial argument of shallow plausibility. Its intellectual basis is *nill*, its moral basis indefensible, its emotional basis unworthy. Opening with a plea for protestants and heretics, the author ranges through a multiplicity of metaphors, comparisons, and illustrations, drawn from the inhabitants of earth, air, and water, and from the domains of art, science, literature, and religion ; through history, ancient and modern, from Roman to Red Man, and finds what he seeks in the conclusion that, as a fit subject for national legislation, it must become a national question. Will it strengthen or weaken the Nation? Will dental laws conduce to our national efficiency? These should be the first points raised in the rational discassion of the question of dental legislation.

THE LEGAL STATUS OF DENTISTS.

Daniel Nason, Esq., New York, in the *Items of Interest*, continues his valuable article on this important topic. The present section deals with the evidence necessary to establish malpractice, the exemption from levy and sale of a dentist's implements, and whether the law contemplates them as the tools of a mechanic or the instruments of a professional man; also his legal rights to recover compensation under various circumstances.

THE RELATIONS OF DENTISTRY AND MEDICINE.

Dr. Geo. W. Williams, Richmond, Ind., in a paper read before the Indiana State Society, as printed in the *Ohio Journal* discusses this question, reaching the following conclusions :

1st. That the degree of Doctor of Medicine is the standard to which dental colleges must attain, to entitle their students to recognition.

2nd. That a formal recognition is not desirable, an independent organization being more effective and giving higher prestige.

3rd. That we are already recognized for all we are worth.

4th. That the dentist of the future will have taken a medical degree and supplemented it by special training for dental practice.

PATENTS.

In a paper published in the *Independent Practitioner*, read before the New Jersey State Dental Society, Dr. B. Holly Smith, Baltimore, discusses the question, "Should a Dentist Patent his Inventions," from the ethical and scientific standpoint, endeavoring to steer wisely between the conservation of the old order of things and the audacity and innovation of the younger element. Reviewing briefly the example and experience of medicine in this matter, and scrutinizing the reasons for the course it has pursued, the writer takes the position that adopted members of a family should not expect to alter its established traditions; if not governed by its principles they are recreant to its practice. But apart from the argument of mere authority, reason suggests that a noble profession, which is a dispenser of blessings and comforts through its beneficent healing powers, should not be degraded to the position of a donkey toiling before the cart of self-profit, or that of a channel through which dollars run, each brother opening a little side pipe, or scooping and scratching from the main, to increase his pile. There should be no profit from pain—no trading in human suffering. We claim to be a scientific profession; the true scientific spirit inquires, investigates, invents, discovers, and scatters the results broadcast to the world; they who patent their inventions lack the very essence and vitality of that spirit. In the discussion of this paper (p. 641), Dr. Atkinson declared that the whole patent system was robbery from beginning to end.

Dr. Haglunst thinks it is the duty of every true man to set his face against the maintenance of the system.

Dr. Thayer, though he has never invented any thing himself, thinks the man who has the ability to invent something valuable, should be protected in his just dues.

In conclusion Dr. Smith said that in throwing ethics to the dogs the profession is robbed of all that makes it a profession, and its members reduced to the level of tradesmen.

Dr. Ivory thought that if Dr. Smith could patent an invention and get a million dollars for it, as Edison had done, he would accept the money and be thankful.

PROSTHETIC DENTISTRY.

A *Cosmos* editorial commends most highly the professional generosity of the clinicians who so unselfishly devoted their time to object-lessons, especially in gold and porcelain crown and bridge-work, which promises so much for the honorable future of prosthetic dentistry through the high degree of manipulative ability and artistic skill requisite to produce these works of art.

PROSTHETIC DENTISTRY.

In the *Ohio Journal* Dr. Haskell describes the greater comparative difficulty of making a comfortable, satisfactory lower denture, discusses the reasons for this, and expresses his willingness to pay for the information how to overcome the difficulty.

CELLULOID.

Dr. F. W. Seabury, Providence, R. I., gives in detail his method of manipulating celluloid, by which he claims all tendency to warp or discolor is overcome and all danger of breaking teeth avoided. By his method the prepared blank is moulded in five minutes, coming out perfectly finished, ready for the mouth without filing or polishing; a process requiring mechanical ability of high order, trained by long experience, and guided by keen judgment, but which saves time and teeth, and insures a high price for artistically perfect dentures.

DISK-CARRIER.

In the clinic of the First District Dental Society, New York, reported in the *Cosmos*, Dr. H. H. Sesson, New York City, exhibited an improved disk-carrier which pushes the rubber-dam out of the way, preventing catching and tearing the dam.

DISK MOISTENER.

Dr. J. P. Carmichael, Milwaukee, Wis., exhibited a mouth-disk moistener which the operator holds in his left hand while operating the engine hand-piece with his right.

*The Independent Practitioner.*

A NEW NITROUS OXIDE BLOW PIPE.

At the union meeting of the Connecticut Valley and Massachusetts Dental Society, Dr. G. F. Harwood, Worcester, Mass., presented to the profession " without let or hinderance as regards either sale or manufacture" a new nitrous oxide blow pipe, which is designed to take nitrous oxide either from the gasometer at low pressure, or from the cylinder at high pressure, a point not covered by any other nitrous oxide blow pipe on the market. The apparatus, while simple and compact, gives perfect regulation and control of a powerful and concentrated flame, suitable for soldering gold and platinum, melting at high temperature, etc.

A NEW HEAD REST

which is presented by Dr. S. G. Stevens, made of wire woven similarly to the woven-wire mattresses.

A CHEAP GAS FURNACE.

Dr. J. E. Stanton presented a simple arrangement for making a gas furnace for continuous gum work, at an expense of not more than three dollars.

